# Circulating miRNA-21 is an innovative biomarker for cardiovascular events in erectile dysfunction patients

**DOI:** 10.3389/fcvm.2024.1301925

**Published:** 2024-03-21

**Authors:** Laura Agulló, Ana Segura, Samanta Ortuño-Miquel, Ana Teresa Brinca, Rosa Micol-Ponce, Vicente Arrarte, María Rosa Ponce, Pau Miró-Martínez, Thomas Zandonai, Ana M. Peiró

**Affiliations:** ^1^ Pharmacogenetic Unit, Clinical Pharmacology Department, Alicante Institute for Health and Biomedical Research (ISABIAL), Dr. Balmis General University Hospital, Alicante, Spain; ^2^ Institute of Bioengineering, Miguel Hernández University, Elche, Spain; ^3^ Andrology Unit, Urology Department, Dr. Balmis General University Hospital, Alicante, Spain; ^4^ Bioinformatics Department, Alicante Institute for Health and Biomedical Research (ISABIAL), Dr. Balmis General University Hospital, Alicante, Spain; ^5^ Health Sciences Research Centre, Faculty of Health Sciences, University of Beira Interior, Covilhã, Portugal; ^6^ Cardiology Department, Dr. Balmis General University Hospital, Alicante, Spain; ^7^ Department of Statistics and Operational Research of the Alcoy Campus of Universitat Politècnica de València, Alicante, Spain; ^8^ Addiction Science Lab, Department of Psychology and Cognitive Science, University of Trento, Rovereto, Italy

**Keywords:** cardiovascular disease, erectile dysfunction, genomic biomarkers, circulating miRNA, miRNA-21

## Abstract

**Introduction:**

It is well-known that circulating microRNAs (miRNAs) play a relevant role in many kinds of diseases by regulating the expression of genes involved in various pathophysiologic processes, including erectile dysfunction (ED) and cardiovascular diseases (CVD).

**Purpose:**

This study aimed to identify the miRNA-21 profile in the blood samples of patients with ED, CVD, and the combination of both pathologies to elucidate the potential function of miRNA-21.

**Methods:**

A total of 45 patients with CVD and/or who underwent the erectile function test were included and divided into the following categories: CVD with ED (cases, *n *= 29) and controls (*n *= 16) with either ED or CVD. Real-time polymerase chain reaction analysis verified the results. miRNA-21 expression was quantified, and informatics analysis was applied to predict the functions of this differentially expressed miRNA-21.

**Results:**

A total of 64% of cases (63 ± 9 years, 66% with severe ED, 56% with CV ejection fraction) first presented ED as the sentinel clinical manifestation. Serum miRNA-21 levels in the control ED were significant, up to 10-fold higher than in the CVD controls and cases. A significant inverse (*p* = 0.0368, *β* = −2.046) correlation was found between erectile function and miRNA-21 levels.

**Conclusions:**

Our study provides comprehensive insights into the functional interaction between miRNA-21 and ED in CVD patients. Its relevance lies in the potential of miRNA as a biomarker to be applied in the cardiovascular predictive medicine field.

## 1 Introduction

Male erectile dysfunction (ED) is notably prevalent in patients diagnosed with cardiovascular diseases (CVD). Both conditions share common risk factors and pathophysiological connections, such as endothelial dysfunction, which is commonly attributed to the age-related degeneration of penile erectile tissue or deficiency in male hormonal levels (1, 2). In fact, ED has been shown to be an early independent factor of future CVD events (3, 4), which suggests an important pathway to initiate preventive measures. Furthermore, although data indicate that pharmacological treatment of ED affects the risk of CVD, adverse events related to sexual dysfunction drugs are a major contributing harbinger of poor adherence to therapy (5). Therefore, screening for ED is essential to prevent CVD by offering an easy prognostic tool (6–9).

For the last 20 years, microRNAs (miRNAs) have been studied in connection to the post-transcriptional regulation of gene expression in critical cardiac physiological and pathological processes (10). miRNAs are short non-coding transcripts of approximately 22 nucleotides. Post-transcriptional gene regulation at the gene expression level is significantly promising as a diagnostic and therapeutic approach for managing various diseases (11, 12). By interrupting protein synthesis, miRNAs can effectively turn genes off and influence many fundamental processes in the body, such as developmental and apoptotic behaviors of cells and cardiac organogenesis. A recent systematic review of 59 articles on experimental studies involving human samples has evidenced that miRNAs can be a powerful gene regulator in CVD (13). Moreover, anomalies in miRNA expression profiles have been noted in the corpus cavernosum of both aging rats with ED and diabetic mice (7), where the upregulation of miRNA-200a can attenuate endothelial function with an impact on atherosclerotic plaques (14). In fact, simvastatin prevented a decrease in isoenzyme eNOS (endothelial nitric oxide synthase) expression while decreasing miRNA-155 levels (15). Overall, the link between miRNA-21 expression and ED and CVD is poorly established in the literature. Some data suggest the possibility of using nanoparticle miRNA-21 delivery to attenuate post-myocardial remodeling by reducing hypertrophy, fibrosis, and cell apoptosis in the remote myocardium (16). Therefore, miRNAs might be implicated in contributing to the pathogenesis of ED and could represent a potential future therapeutic strategy.

This clinical study aims to analyze the expression levels of miRNA-21 in the serum samples of patients with CVD and/or ED to elucidate the intricate pathways underlying endothelial dysfunction.

## 2 Materials and methods

### 2.1 Study design

A prospective study was designed and conducted at the Cardiology Rehabilitation Centre and Andrology Unit in the Department of Health of Dr. Balmis General University Hospital of Alicante, Spain from March 2022 to June 2023. The study was approved by the Research Ethics Committee (Protocol code: PI2022-114). After obtaining approval from the Ethics and Research and Development committee, 84 men with CVD and/or ED were recruited from the Cardiac Rehabilitation Centre and Andrology Unit of the Department of Health of Dr. Balmis General University Hospital of Alicante. Among them, those with blood samples were finally included (*n *= 45). All the participants received detailed information about the study design and its objectives from their healthcare provider, and written informed consent was duly obtained. Inclusion criteria were the following: patients older than 18 years, with a diagnosis of ED [“erectile function” domain of the International Index of Erectile Function (IIEF-EF) questionnaire ≤25], and/or being assisted in the Cardiology Unit. Individuals were excluded if they had been previously diagnosed with conditions affecting the brain, spinal cord, or pelvic nerves (e.g., multiple sclerosis, multisystem atrophy, spinal cord injury, and tumors), conditions affecting the cauda equina (such as prolapsed intervertebral discs or tumors), and diseases affecting the parasympathetic nerves within the pelvis. In addition, individuals who had received extensive surgery to the pelvis or abdomen and individuals diagnosed with conditions such as chronic renal failure, hyperprolactinemia, hypogonadism, smooth muscle dysfunction, and Peyronie's disease were also excluded.

Professional nurses interviewed all the participants who attended cardiac rehabilitation programs using a standardized interview and a structured questionnaire that covered socio-demographic and clinical characteristics. High-risk CVD individuals were included if they had a history of myocardial infarction or angina. Following the collection of questionnaire data, the patients who mentioned any sexual dysfunction were referred to the Andrology Unit for diagnosis and treatment. The age of the patients ranged from 37 to 76 years, and none were taking hormone medication (supplementation or deprivation) or any phosphodiesterase type 5 (iPDE5) inhibitors before being included in the present study. Prescriptions were made as usual in the Andrology Unit for ED management and included iPDE5 (sildenafil, tadalafil, avanafil or vardenafil). A health professional also completed a medical details questionnaire. All patients underwent a thorough medical examination, including a review of their medical and drug history.

### 2.2 Clinical outcomes

To evaluate male sexual function, particularly the presence or absence of ED, the IIEF questionnaire was utilized. The IIEF comprises 15 items that are categorized into five domains of sexual function: erectile function (EF), orgasmic function, sexual desire, satisfaction with sexual intercourse, and overall satisfaction. In this questionnaire, higher scores indicate lower dysfunction levels. The domain that assesses erectile function (IIEF-EF) comprises six questions with a maximum score of 30 and serves as a reliable tool for categorizing the degree of ED as severe (1–10), moderate (11–16), mild (17–25), and normal or no dysfunction (≥26) (17).

In addition, patients were instructed to fill out a questionnaire related to their sexual quality of life, namely, the Sexual Life Quality Questionnaire (mSLQQ). The mSLQQ is a comprehensive tool that allows patients and their partners to compare their experiences before the onset of ED with their experiences since treatment onset. This questionnaire comprises 10 assessment items that cover aspects such as frequency and duration of sexual activity, ease of penetration, ease of achieving orgasm, initiation of sexual encounters, pleasure of anticipation, carefree feelings during sexual activity, pleasure derived from orgasm, overall satisfaction, and partner's satisfaction (18, 19).

The psychological status was assessed in accordance with the Hospital Anxiety and Depression Scale (HADS), which scores from 0 to 21. The scores are classified as normal (<7), probable (8–10), and case (>11) (20, 21).

At the time of inclusion, the cardiovascular ejection fraction was recorded. According to the American Heart Association, an ejection fraction of about 50%–70% is categorized as normal, a mild fraction is 41%–49%, and a reduced ejection fraction is 40% or lower.

### 2.3 Sample collection and plasma processing

Samples from patients included in this study were provided by the BioBank ISABIAL, adhered to the Spanish National Biobanks Network and integrated in the Valencian Biobanking Network and they were processed following standard operating procedures with the appropriate approval of the Ethical and Scientific Committees. Blood samples were collected in vacutainers containing EDTA. Then, samples were immediately centrifuged for plasma separation at 3,000×*g* for 10 min. After phase separation, plasma was collected and divided into two aliquots, quickly frozen, and preserved at −80°C until the RNA isolation process. The total RNA from blood samples was isolated using the miRNeasy Serum/Plasma kit (Qiagen, Dusseldorf, Germany) according to the manufacturer's instructions. The purity and concentration of the RNA samples were assessed spectrophotometrically by a NanoDrop ND-2000c (Thermo Fisher Scientific, Inc., Wilmington, DE, USA).

### 2.4 miRNA quantitative real-time PCR

First-strand complementary DNA (cDNA) synthesis was carried out with 10 ng of total RNA from each sample with the TaqMan MicroRNA Reverse Transcription kit (Applied Biosystems, Carlsbad, CA, USA) following the manufacturer's protocol. cDNA was amplified using TaqMan Universal MasterMix II (Applied Biosystems, Carlsbad, CA, USA) in a QuantStudio 12K Flex Real-Time PCR system (Applied Biosystems).

Real-time quantitative PCR (RT-qPCR) assays were performed in triplicate on diluted cDNA templates using specific TaqMan MicroRNA assays for hsa-miR-21-5p (Assay ID: 000397) and RNU6B (Assay ID: 001093) (Thermo Fisher Scientific) according to the manufacturer's instructions. The relative quantification of the miRNA expression levels was performed according to the 2-ΔΔCt method (22). Quantitative results were obtained using RNU6B as an endogenous reference for normalization.

### 2.5 Data analysis

The Shapiro–Wilk normality test was performed for fewer than 50 samples. In contrast, the Kolmogorov–Smirnov normality test was used to determine whether to apply a parametric or non-parametric test for comparisons. The quantitative data are presented as the mean ± standard deviation (SD). The categorical variables are expressed as total numbers and percentages. Demographic and clinical data were compared using either the *χ*^2^ or Fisher's exact test for the categorical variables, depending on the sample size of groups, and the *t*-test or Mann–Whitney *U* test for the continuous variables, depending on their distribution. When more than two groups were involved, ANOVA or Kruskal–Wallis tests were used for the continuous or categorical variables, respectively. In all cases, multiple testing was adjusted by the Bonferroni correction method. Statistical analyses were performed with the R software (v 4.3.1), and *p* < 0.05 was considered statistically significant.

## 3 Results

All participants with CVD who reported sexual dysfunction in the Cardiology Rehabilitation Program and/or presented with ED were referred to the Andrology Unit. ED was confirmed in 29 cases (CVD + ED) through a clinical routine. The mean age of patients with both pathologies was 63 ± 9 years, with the onset of CVD or ED occurring at 53 ± 12 or 54 ± 18 years old, respectively, as presented in Table 1. In the Cardiology Unit, seven patients with only CVD were included as controls, with a mean age of 50 ± 5 years and a mean age of CVD onset of 51 ± 4 years. In the Andrology Unit, nine patients with ED but without CVD were included as controls, with a mean age of 60 ± 12 years and a mean age of ED onset of 54 ± 12 years.

**Table 1:** Socio-demographic and clinical data of the patients in three groups: (1) controls with ED; (2) controls with CVD; and (3) cases with both pathologies.

	Controls with ED	Controls with CVD	Cases with ED + CVD
Patients [% (*n*)]	20 (9)	16 (7)	64 (29)
Age [years, mean (SD)]	60 (12)*	50 (5)	63 (9)**
Onset age of CVD [years, mean (SD)]	NA	51 (4)	53 (12)
Onset age of ED [years, mean (SD)]	54 (12)	NA	54 (18)

NA, not applicable.

* *p* < 0.05.

** *p *< 0.005.

All participating cases with CVD and ED showed impacted erectile function (IIEF score: 34 ± 15; IIEF-FE score: 11 ± 6) with no impact on mental health (anxiety and depression), and their mean body mass index (BMI) was 24 ± 11 kg/m^2^ (18.5–25 kg/m^2^, falling within the normal weight range), as presented in Table 2.

**Table 2:** Clinical data of the patients in the group of cases with ED and CVD.

	Cases with ED + CVD (*n *= 29)
IIEF	34 (15)
IIEF-FE	11 (6)
mSLQQ	24 (7)
HADS-depression	3 (4)
HADS-anxiety	5 (5)
BMI	24 (11)

IIEF: 0–57 scores (normal >30); IIEF-FE: 0–30 scores (normal >26); HADS: 0–21 scores; BMI: 18.5–25 kg/m^2^ indicates normal weight and <25 kg/m^2^ indicates being overweight.

Values are presented as mean (SD).

The miRNA-21 expression data of the controls (ED, CVD) and cases (both pathologies) are described in Supplementary Table S1. Significant differences in the miRNA-21 expression levels were obtained among the three groups (ED vs. ED + CVD; CVD vs. ED + CVD; ED vs. CVD; *p* < 0.001). Linear regression analysis showed an inverse association between miRNA-21 expression levels and ED prevalence (*p *= 0.0368, *β *= −2.046).

**Figure 1:**
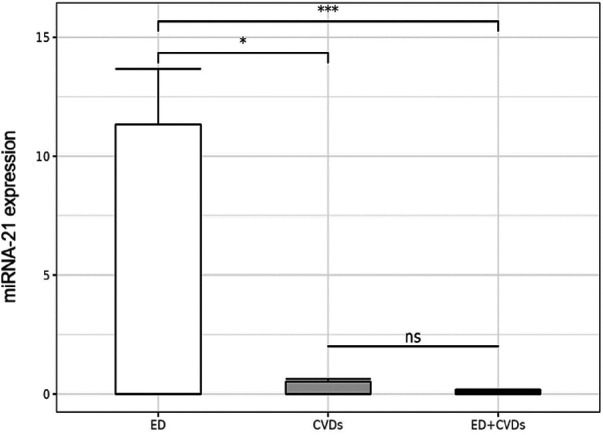
Relative microRNA-21 expression levels in the three patient groups. *p < 0.05; ***p < 0.0005; ns: not significant.

Also, linear regression analysis revealed significant differences between the ED group and patients with CVD and both pathologies. As depicted in Figure 1 and Supplementary S1, patients with CVD presented significantly lower miRNA-21 expression levels compared to the other two groups (*p *= 0.0368, *β *= −2.046), whereas no significant differences were found between the patients with CVD and those with CVD + ED (*p *= 0.5).

**Figure 2:**
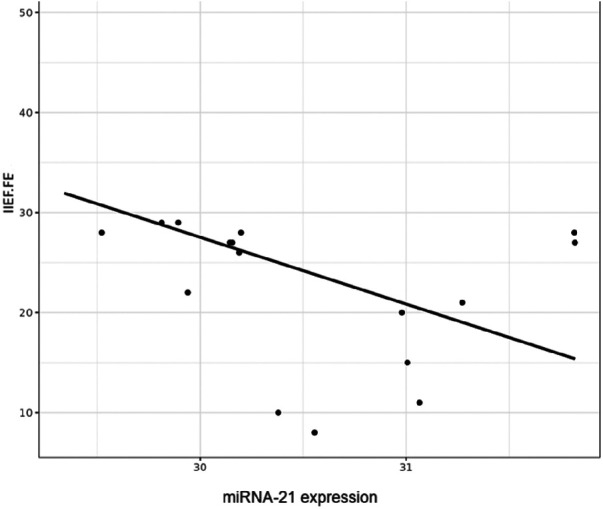
Correlation figure of the relative microRNA-21 expression levels and the IIEF-FE (0–30; normal >26).

Although the numerical correlation was not very high (*r*^2^ = −0.21), correlation analyses showed a negative association trend between the relative miRNA-21 expression and the IIEF-FE questionnaire results, with the highest miRNA expression levels observed in those with the highest degree of ED (see Figure 2).

## 4 Discussion and conclusions

The results reported an inverse correlation between miRNA-21 expression and erectile function. One important finding was the significantly elevated serum level of miRNA-21 in ED patients with CVD compared to those with ED alone. This represents an excellent opportunity for conducting general screening in cardiovascular risk patients.

A recent publication defines the theranoMiRNA term by highlighting the utility of miRNAs in diagnosis and treatment (23). Considering that CVD are the leading cause of death in the world, which is even higher in women (24), the identification of theranoMiRNAs can be essential, and future research should include women among participants. Previous evidence has demonstrated the upregulation of four miRNAs in ED rat penile tissue compared to young rats using microarray analysis (25). Moreover, miRNA-21 could be involved in various cardiomyopathies, as its expression levels undergo significant changes in both cardiac tissue and circulation to offer cardiac protection following heart injury. Our results revealed that miRNA-21 showed significant differences between ED controls and male CVD patients. However, other studies with different populations have demonstrated that individuals with acute heart failure exhibited high plasma miRNA-21 levels from admission to hospital discharge, which subsequently decreased during the clinical compensation period (26). In addition, similar time-dependent fluctuations in circulating miRNA-21 expression have also been observed in patients with post-myocardial infarction (27). Further studies are needed to reveal any potential link between miRNAs and circRNAs or miRNAs (28) correlated with prognosis and predictive value for heart failure (11) in CVD patients across different comorbidities (29, 30) and populations, as well as therapeutic agents in treatment (31, 32), including in female CVD patients. Studies have also investigated the potential use of circulating serum miRNA and fecal miRNA expression as non-invasive markers for early detection (33), which might aid in guiding therapeutics (34). Moreover, in our sample, sexual symptoms should be considered a harbinger for cardiovascular screening, given the opportunity to check ED as a symptom that may help in better managing patients, their comorbidities, therapy, and complications (35, 36). ED, serving as a sentinel marker, could be the first clinical presentation of subclinical endothelial dysfunction disease, affecting even younger men than those in our sample. Our data support using miRNAs as emerging prognostic markers to further understand the cardiovascular risk in men. Thus, miRNA-21 could be potentially involved in the progression of endothelial damage in male ED patients in the future. This is important because it demonstrates that ED offers physicians a unique opportunity to see the possible future cardiovascular health of their patients and to conduct specific evaluations for CVD.

### 4.1 Limitations

It is well known that observational studies can be affected by several biases. First, the sample size was limited because it was derived from a “convenience sample” from a single center. Dietary habits, lifestyle factors, and limitations in detection methods may influence these findings and their implications for cardiovascular risk. Hence, it is imperative to interpret our results by considering various backgrounds, the multifaceted nature of ED, and the necessity to replicate these results in diverse ethnic populations by including a group of healthy patients without any disease. Furthermore, antihypertensive drug use, depression, and anxiety are the main factors to affect ED. Other sources of error may relate to the complexity of questions or the process required to access the desired information from memory. Only in cases was complete clinical information provided. Future studies will include a complete set of clinical information for both controls and cases.

It is well established that the expression of miRNAs is tightly controlled and is tissue-, developmental stage-, and disease-specific (37). However, we are still unclear about how miRNA-21 expression is regulated in diseased hearts. In future studies, a combination of bioinformatics tools and computational analyses will be needed for the miRNA-21 target gene study.

Briefly, our results strongly support that miRNA expression is higher in ED than in CVD patients associated with erectile function in our study population. Furthermore, future studies need to evaluate the potential use of miRNAs as non-invasive biomarkers to prioritize patients with ED for CVD screening.

## Data Availability

The original contributions presented in the study are included in the article/Supplementary Material, further inquiries can be directed to the corresponding author.

## Supplementary material

Supplementary Figure S1, Supplementary Table S1 Enhanced plot of Figure 1: relative expression levels of miRNA-21 among patients with cardiovascular disease (CVD) and patients with both pathologies (ED + CVD); ns: not significant. MicroRNA-21 and RNU6B (endogenous control) expression. Data were obtained by qPCR to determine the relative expression levels of miRNA-21 by the 2-ΔΔCt method (22) in three groups of patients: (1) controls with erectile dysfunction (ED); (2) controls with cardiovascular diseases (CVD); and (3) cases with both pathologies.
