# Supplementary material for: Circulating miRNA-21 is an innovative biomarker for cardiovascular events in erectile dysfunction patients
Source: Front Cardiovasc Med. 2024 Mar 21;11:1301925. doi: 10.3389/fcvm.2024.1301925 (PMC10991794; doi:10.3389/fcvm.2024.1301925)
Supplement: Supplementary Figure S1, Supplementary Table S1 — Enhanced plot of Figure 1: relative expression levels of miRNA-21 among patients with cardiovascular disease (CVD) and patients with both pathologies (ED + CVD); ns: not significant. MicroRNA-21 and RNU6B (endogenous control) expression. Data were obtained by qPCR to determine the relative expression levels of miRNA-21 by the 2-ΔΔCt method (22) in three groups of patients: (1) controls with erectile dysfunction (ED); (2) controls with cardiovascular diseases (CVD); and (3) cases with both pathologies. [file Datasheet1.pdf]

**Supplementary material**

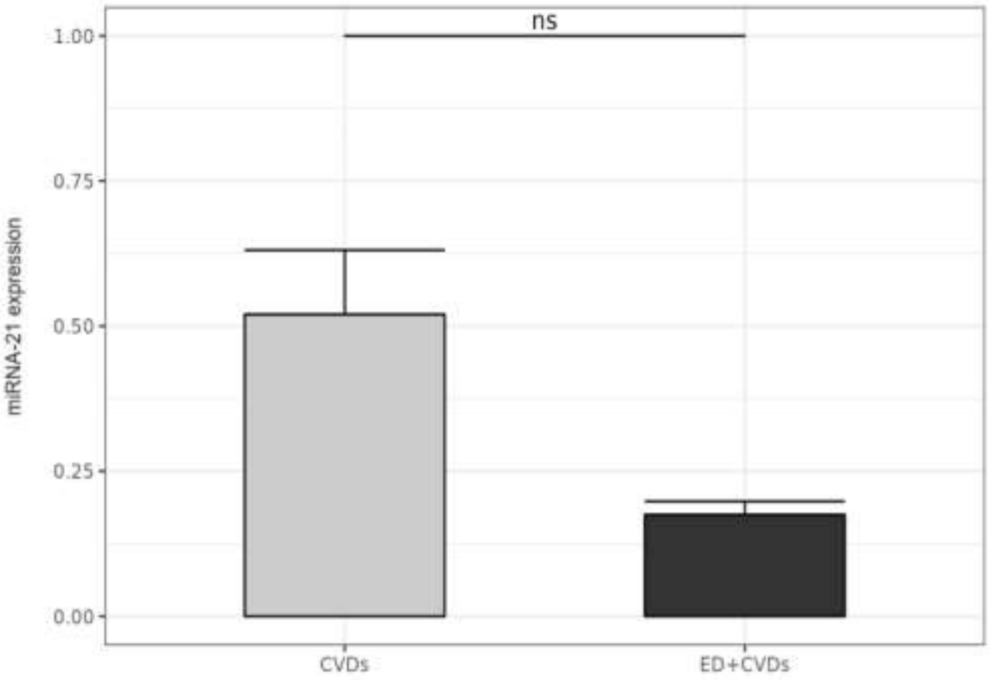


**Figure S1.** Enhanced plot of Figure 1: relative expression of miRNA-21 between patients with cardiovascular disease (CVDs) and patients with both pathologies (ED + CVDs); ns: not significant.

**Table S1.** MicroRNA-21 and RNU6B (endogenous control) expression. Data obtained by qPCR to determine the relative expression of miRNA-21 by $2^{-\Delta\Delta Ct}$ method [22], of the patients in group 1 – control erectile dysfunction (ED); 2 – control cardiovascular diseases (CVDs); 3 – cases with both pathologies.

| | **Control Erectile Dysfunction (ED)** | **Control Cardiovascular diseases (CVDs)** | **Cases ED + CVDs** |
|---|---|---|---|
| **miRNA-21 expression (mean (SD))** | 23 (6) | 27 (3) | 30 (1) *+ |
| **RNUB6 expression (mean (SD))** | 31 (2) | 33 (1) | 35 (1) |

*: $p < 0.05$ between the control erectile dysfunction patients (ED) *vs*. cases; +: $p < 0.05$ between the control cardiovascular disease patients (CVDs) *vs*. cases.
